# Microvessel density is as a prognostic factor in canine cutaneous mast cell tumors

**DOI:** 10.1186/1753-6561-7-S2-P62

**Published:** 2013-04-04

**Authors:** Daniella FL Barbosa, Lidia H Pulz, Nilton P Santos, Silvia Regina Kleeb, José Guilherme Xavier, José Luiz Catão-Dias, Ricardo F Strefezzi

**Affiliations:** 1 Department of Veterinary Medicine, Faculty of Animal Sciences and Food Engineering, University of São Paulo, Pirassununga/SP, Brazil; 2 Department of Pathology, Faculty of Veterinary Medicine and Animal Sciences, University of São Paulo, São Paulo/SP, Brazil; 3 Course of Veterinary Medicine, Paulista University, São Paulo/SP, Brazil; 4 Course of Veterinary Medicine, Metodista University, São Bernardo do Campo/SP, Brazil

## Background

Mast cell tumors represent almost 25% of canine skin neoplasms. These tumors are classified in three grades of differentiation, based on histological features. However, this grading system is a method based on subjective parameters, which generate intra- and interobserver variations. The microcirculation is an important feature to the primary tumor expansion, dissemination and metastasis, and there are essential evidences that increasing microvessel density is associated with short survival disease-free intervals. The purpose of the present study was to verify the prognostic value of the intratumoral microvessel density (IMVD) in a set of canine cutaneous mast cell tumors.

## Materials and methods

Twenty-nine canine cutaneous mast cell tumors were subjected to immunohistochemical analysis using a rabbit polyclonal antibody anti-human von Willebrand Factor. Subsequently, the IMVD was determined by the average number of vessels in 5 low-power fields.

## Results

The average IMVD was 9.1 vessels/field for grade I mast cell tumor cases, 14.1 for grade II and 17.2 for grade III. There was no statistically significant differences between histopathological grades with regard to IMVD (p=0.0881). Nevertheless, IMVD was significantly higher (p=0.0362) in dogs which died due to the mast cell tumor. After the identification of a cutoff point by ROC curve analysis (12.6 vessels/field), cases were divided into two groups. Survival analysis showed that mast cell tumors with higher IMVDs had a worse prognosis (p=0.0064), with median survival of 751 days.

## Conclusions

The IMVD is a trustworthy prognostic factor, indicating the post-surgical survival in cases of canine cutaneous mast cell tumors.

## Financial support

FAPESP (grant no. 2010/05094-5) and CNPq (PIBIC 2011-459).

